# Prevalence-Aware Networks of Social Activity and Community Facility Use Among Older Adults in the Republic of Korea: Comparisons by Age, Residence, and Sex

**DOI:** 10.3390/geriatrics11050150

**Published:** 2026-10-20

**Authors:** Ha-Na Kim, Sang-Hwa Lee, Jong-Bae Choi

**Affiliations:** 1 Department of Occupational Therapy, Sangji University, 83 Sangjidae-gil, Wonju-si 26339, Republic of Korea; hannah@sangji.ac.kr; 2 Mapo-gu Dementia Center, 3rd Floor, 50 Daeheung-ro 24-gil, Mapo-gu, Seoul 04123, Republic of Korea; sh201024@naver.com; 3 Department of Occupational Therapy, Chosun University, 309 Pilmun-daero, Dong-gu, Gwangju 61452, Republic of Korea

**Keywords:** social participation, community facilities, older adults, network analysis, subgroup analysis, survey weights

## Abstract

**Background/Objectives**: Older adults engage in diverse activities and use community facilities, but how these forms of participation connect is unclear. We examined these connections and whether the findings depended on how common each activity or facility was. **Methods:** Data from 10,078 participants in the 2023 National Survey of Older Koreans were used to estimate 13-node domain–facility and 20-node specific activity–facility networks with survey-weighted φ coefficients and 1000 stratified cluster-bootstrap replicates. Leisure–leisure pairs were excluded from the specific network because only two main leisure activities could be reported. Sensitivity analyses used sign-specific attainable φ limits, normalized φ, covariate-adjusted partial correlations, semantic-overlap exclusions, and subgroup log-odds-ratio contrasts. **Results:** The domain network retained 27 positive and one negative edge; the specific network retained 34 positive and one negative edge among 162 estimable pairs. Original and φ-limit-normalized strength rankings correlated at ρ = 0.852 (domain) and 0.968 (specific), but public cultural facilities moved from first to fourth and third, respectively, with socializing groups ranking first in both normalized networks. Eighteen of 28 domain edges and 22 of 35 specific edges were retained with the covariate-adjusted partial-correlation criterion. Public cultural facilities had the largest strength point estimates in both primary networks, although several leading nodes were not statistically distinguishable; the prominence of education/learning was also sensitive to broad removal of definitionally overlapping links. Subgroup strength rankings were similar, whereas several differential φ edges were not confirmed by log-odds-ratio contrasts. **Conclusions:** The networks identify disproportionate co-participation patterns rather than prevalence-free or causal relationships. Overall rank agreement under normalization was higher in the specific than the domain network, but neither preserved the leading node. Marginal constraints, item overlap, and the association measure affected interpretation.

## 1. Introduction

Social participation encompasses activities through which individuals interact with others and contribute to community life. In later life, such participation is relevant not only to social contact but also to identity, role continuity, belonging, and access to informational and practical resources [1,2,3,4,5].

Most gerontological studies operationalize participation as the presence, frequency, or summed number of activities. These measures are useful for estimating exposure or dose, but they do not show whether activities tend to be combined for the same people. Activity-profile and latent-class studies have demonstrated that older adults organize participation into heterogeneous repertoires rather than a single continuum from inactive to active [6,7,8].

Community facilities are a second component of this ecology. Senior centers, welfare centers, schools, and public or private cultural facilities provide physical settings, programs, and opportunities through which participation can occur. Neighborhood characteristics and the availability of suitable locations are associated with social functioning and activity participation in later life [9,10].

The health relevance of participation is well established. Longitudinal studies have linked social activity to lower risks of incident disability, mortality, frailty progression, and dementia, while systematic reviews indicate potential benefits for cognitive functioning [11,12,13,14].

Nevertheless, an important structural question remains: which activities and facilities are disproportionately connected after accounting for how common each one is? Interpersonal network studies describe relationships among people, whereas an activity network treats activities or facilities as nodes and within-person co-participation as edges. The latter is an affiliation-based network: it describes the organization of participation at the population level and should not be interpreted as a network of interpersonal ties [15,16,17,18].

A raw co-occurrence count is problematic for this purpose because a common activity has more opportunities to co-occur with every other node. Raw weighted degree is therefore algebraically tied to node prevalence. Binary φ coefficients standardize the 2 × 2 covariance by the marginal variances and reduce this scale dependence, but they are not prevalence-free: unequal marginals restrict the largest attainable positive or negative coefficient. Accordingly, φ-based networks should be interpreted together with node prevalence, attainable φ limits, and sensitivity analyses rather than described as fully prevalence-adjusted.

Participation opportunities are unlikely to be organized identically across older adults. Social roles, mobility, local service availability, and accumulated activity histories differ by age, place, and sex. Studies using activity profiles or community-care samples have reported corresponding differences in the type and breadth of participation [19,20,21]. A pooled national network may therefore describe the common structure while overlooking subgroup-specific routes through which activities and facilities are combined.

This study examined whether the apparent prominence of social activities and community facilities reflected population reach or disproportionate relational embeddedness. We compared raw co-occurrence with survey-weighted φ networks, evaluated the marginal constraints on φ, and compared network structure by age, residence, and sex. We also assessed covariate-adjusted partial associations, alternative log-odds-ratio subgroup contrasts, centrality differences, and sensitivity to potential semantic double-coding.

## 2. Materials and Methods

### 2.1. Study Design and Data Source

This was a secondary cross-sectional analysis of the public-use 2023 National Survey of Older Koreans. The survey is a statutory national survey conducted every three years under Article 5 of the Welfare of Senior Citizens Act. It used stratified multistage cluster sampling based on census survey areas and households, with face-to-face computer-assisted interviews of community-dwelling adults aged 65 years or older. The original survey included 10,078 respondents and was approved by the Korea Institute for Health and Social Affairs Institutional Review Board (KIHASA IRB No. 2023-078) [22].

All 10,078 respondents were retained because the activity, facility, weight, and survey-area variables required for the network analyses were complete. The sample included 977 survey areas. A standardized sample weight (s_wgt1; sum = 10,078) was used for network estimation, and a population post-stratification weight (f_wgt1; sum = 9,499,933) was used to describe the represented population. Because the two weights differed only by a constant multiplier, they produced identical weighted proportions and φ coefficients.

### 2.2. Node Definitions

All nodes were binary. The domain–facility network comprised seven activity domains and six facility-use indicators. The exact Korean questionnaire wording and English translations are provided in Supplementary Table S1. Culture/leisure (D3), education/learning (D4), and volunteering (D6) asked about participation during the past year. Clubs, socializing groups, and political/social organizations (D5_1–D5_3) were coded as present when any participation frequency was reported for the past year. Religious activity was coded as present when D7_1 indicated any religion-related activity during the past year.

The facility question (D9) asked whether any of the following facilities had been used during the past year: a senior center, senior welfare center, social/disability welfare center, senior school/class, public education/leisure/cultural facility, or private education/leisure/cultural facility. These items identify facility use but not which activity occurred there. Importantly, the education follow-up (D4-2) explicitly lists several of the same settings as learning venues; a single episode may therefore be represented by both an activity and a facility node.

The specific activity–facility network comprised 20 nodes. The aggregate leisure node was replaced by eight categories derived from either of the two main leisure activities reported in D3-1: arts attendance, arts participation, watching sports, playing sports, tourism, hobbies/recreation, rest activities, and social/other leisure. Absence means only that a category was not among the two main reported activities. Because the two-response cap mechanically couples leisure categories, all 28 leisure–leisure pairs were excluded from estimation, density calculations, and the multiple-testing family; 162 estimable pairs remained.

### 2.3. Descriptive Analysis

Unweighted counts and weighted percentages were calculated for demographic characteristics and all network nodes. Counts therefore represent actual respondents, whereas percentages represent the older Korean population after application of the survey weights. For leisure subactivities, percentages refer to being named among the two main reported leisure activities.

### 2.4. Network Estimation and Edge Retention

For each estimable pair of binary nodes, a survey-weighted 2 × 2 table was constructed and the weighted φ coefficient was calculated. φ is a standardized marginal association, not a conditional edge and not a fully prevalence-free measure. Therefore, for each pair, we also calculated the sign-specific maximum attainable |φ| from its weighted marginal prevalences and the normalized coefficient φ/φ-limit. The normalization denominator was the positive upper bound for a positive observed φ and the magnitude of the negative lower bound for a negative observed φ. For culture/leisure and rest activities, we additionally summarize both directional bounds across all estimable pairs.

Uncertainty was estimated with 1000 stratified cluster-bootstrap replicates. Survey areas were resampled with replacement within 30 observed province × urban/rural strata, preserving the number of sampled areas per stratum. Two-sided *p* values based on bootstrap standard errors were adjusted via the Benjamini–Hochberg procedure across all estimable edges. A primary edge required q < 0.05, a percentile 95% bootstrap interval excluding zero, and |φ| ≥ 0.10. The effect-size cutoff followed the conventional small-correlation benchmark [23]; it was a screening rule, not a clinical-importance threshold or a prevalence-neutral criterion.

Sensitivity analyses used an inclusive |φ| ≥ 0.05 threshold, φ-limit-normalized strength, and weighted partial correlations after residualizing every node on age, sex, education, household-income quintile, self-rated health (with a missing indicator), and urban/rural residence. The partial-correlation analysis was used as a covariate-adjusted sensitivity analysis and was not described as an Ising or conditional graphical model. We also removed prespecified pairs with potential semantic overlap. The targeted analysis removed education/learning–senior welfare center and education/learning–senior school; an expanded analysis additionally removed education links to public/private cultural facilities and, in the specific network, arts participation–education, hobbies/recreation–education, playing sports–clubs, and social/other leisure–socializing groups.

### 2.5. Network Measures and Stability

Positive strength was the sum of retained positive φ coefficients incident on a node. Activity–facility bridge strength was the sum of retained positive coefficients linking an activity node to a facility node, adapting the bridge-strength concept to the predefined activity and facility node groups [24]. Degree was the number of retained positive edges. Negative edges were reported separately and were not added to positive strength or bridge strength. Signed expected influence was not used.

Raw co-occurrence strength was included only as a demonstration of its algebraic dependence on prevalence. Centrality uncertainty was obtained across the 1000 bootstrap estimates on the fixed positive primary topology. Rank stability was summarized with bootstrap Spearman correlations. Pairwise bootstrap difference tests compared node strengths; overlap between two separate confidence intervals was not treated as a formal significance test [25].

### 2.6. Subgroup Network Comparisons

Subgroup networks were estimated for ages 65–74 versus ≥75 years, urban versus rural residence, and men versus women. The 75-year cutoff follows the conventional distinction between early older age (65–74 years) and later older age (≥75 years) [26]; it was used to facilitate an interpretable subgroup comparison, not as a clinical threshold. The same stratified cluster-bootstrap draws were applied to both groups in each contrast. A differential edge had to meet the primary retention rule in at least one subgroup, have |Δφ| ≥ 0.05, q < 0.05, and a percentile interval excluding zero. Subgroup-specific prevalences and attainable φ limits were reported. As a marginal-invariant sensitivity analysis, weighted log odds ratios were compared with the same paired bootstrap draws and BH-FDR control. Facility–facility edges were examined separately from activity–facility edges.

Overall similarity was summarized descriptively by Spearman correlation between all subgroup edge weights. To compare node connectivity without changing the set of edges from one subgroup to another, subgroup strength and bridge strength were recalculated on the positive edges retained in the pooled primary network. This common-topology approach separated differences in edge magnitude from differences introduced by subgroup-specific thresholding. Strength-rank correlations were treated as descriptive measures. Both the domain and specific networks were examined.

### 2.7. Software and Reproducibility

Analyses were conducted in Python 3.12 using NumPy 2.3.5, SciPy 1.17.0, and Matplotlib 3.10.8. The random seed was 20260727. Supplementary Code S1 reads the public SPSS file, documents node construction, reproduces the stratified cluster bootstrap, and generates the reported tables and figures.

### 2.8. Ethics

The analysis used a deidentified public-use file and involved no contact with participants. The original survey protocol was approved by the KIHASA Institutional Review Board (No. 2023-078), and the original survey reports voluntary participant consent [22].

## 3. Results

### 3.1. Participant Characteristics

The sample included 10,078 adults aged between 65 and 103 years (weighted mean age, 74.14 years); 56.1% were women. Approximately 73.9% lived in urban areas, 60.0% had a spouse, 38.2% had completed high school or higher education, and 39.0% were currently employed. The weighted distribution of household income was approximately 20% in each quintile by construction. Good or very good self-rated health was reported by 42.7% of participants (Table 1).

### 3.2. Prevalence of Activities and Facility Use

Culture/leisure was the most prevalent activity domain (81.3%), followed by socializing groups (54.2%) and religious activities (33.1%). Volunteering was reported by 2.5% of the weighted population. Senior centers were the most frequently used facility (26.5%), followed by senior welfare centers (9.6%) and public cultural facilities (7.0%). Among the two main reported leisure activities, rest activities were most common (62.5%), followed by social/other leisure (33.0%) and hobbies/recreation (29.1%) (Table 2). The mean number of leisure activities named per leisure participant was 1.74.

### 3.3. Prevalence Confounding in Raw Co-Occurrence Networks

Prevalence correlated −0.077 with positive φ-based strength in the domain network and 0.158 in the specific network; the latter was a small positive rather than a near-zero negative association. Moreover, 6 of 78 domain pairs and 10 of 162 estimable specific pairs could not attain |φ| = 0.10 in the direction of the observed association given their marginals, demonstrating that φ remained constrained by the marginal prevalences (Table 3).

### 3.4. Domain–Facility Network

**Figure 1:**
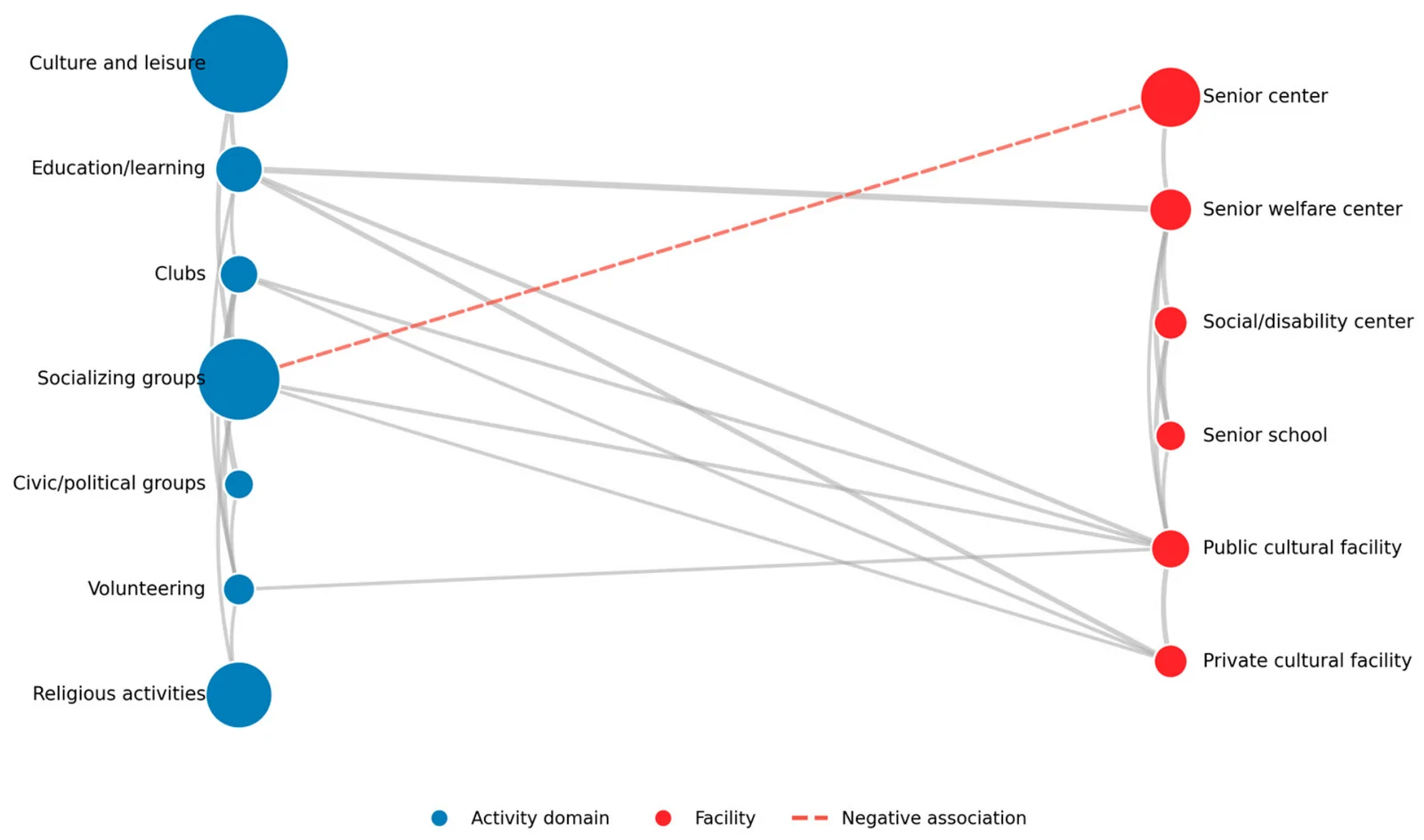
Domain–facility participation network. Note. Node size represents weighted prevalence. Edge width represents |weighted φ|. Gray solid edges are positive; red dashed edges are negative. Shown edges satisfy q < 0.05, a bootstrap interval excluding zero, and |φ| ≥ 0.10.

The 13-node network retained 27 positive and one negative edge (density = 0.359; Figure 1). The strongest edge linked education/learning with senior welfare center use (φ = 0.309, 95% CI 0.271–0.350). Other prominent edges linked clubs with political/social organizations (φ = 0.283), senior welfare centers with senior schools (φ = 0.227), public with private cultural facilities (φ = 0.222), and education/learning with private cultural facilities (φ = 0.216).

The only negative primary edge linked socializing groups with senior center use (φ = −0.120, 95% CI −0.155 to −0.085). Its weighted joint prevalence was 11.7%; the negative coefficient indicates lower joint occurrence than expected from the marginals, not an absence of joint participation.

Public cultural facilities had the largest positive-strength point estimate (1.213), followed descriptively by education/learning (1.107), senior welfare centers (1.018), clubs (0.976), and socializing groups (0.888). Education/learning had the largest activity-side bridge-strength estimate (0.712), equal to the sum of its three retained facility links (0.309 + 0.216 + 0.186). Pairwise activity–facility φ values for unchanged nodes were identical in both networks; hence, the activity-side bridge values of unchanged activity nodes were identical. Facility-side bridge strength was not generally invariant. For public cultural facilities, facility–facility edges contributed 0.631 to its 1.213 domain strength (52.0%) and 0.631 to its 1.343 specific-network strength (47.0%); the remaining contributions were activity–facility links (Table 4). Senior welfare center bridge strength happened to remain 0.309 because neither aggregate leisure nor any leisure category had a retained positive edge with that facility. Bootstrap difference tests showed that public cultural facility strength did not differ from education/learning (difference 0.106, 95% CI −0.063 to 0.262), but exceeded senior welfare center strength (difference 0.195, 95% CI 0.049–0.337). Education/learning did not differ from senior welfare centers (difference 0.089, 95% CI −0.057 to 0.233).

### 3.5. Specific Activity–Facility Network

**Figure 2:**
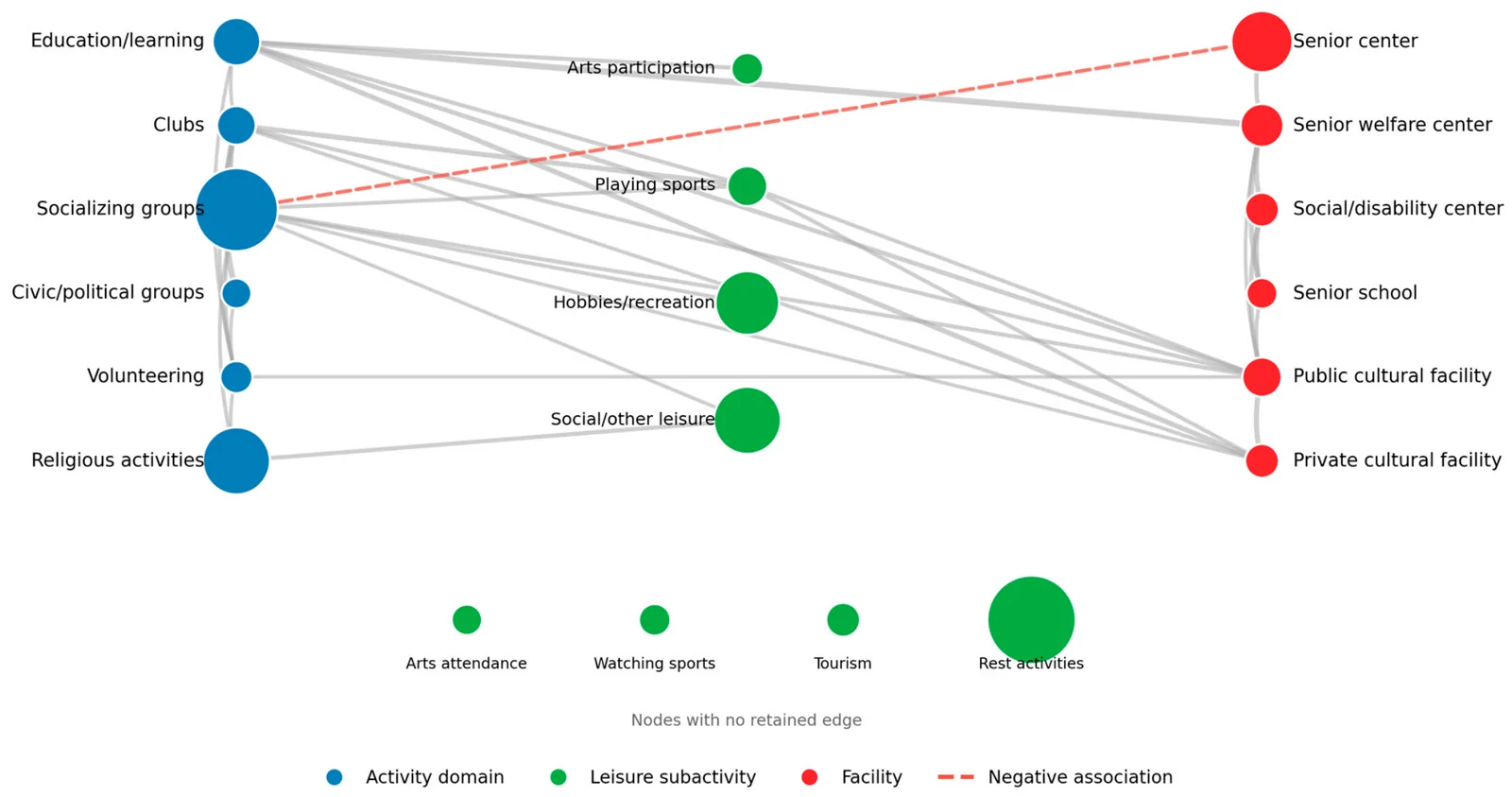
Specific activity–facility participation network. Note. The aggregate leisure node and all leisure–leisure pairs are excluded. Leisure-category nodes identify categories named among at most two main activities. Shown edges satisfy q < 0.05, a percentile interval excluding zero, and |φ| ≥ 0.10.

After excluding all leisure–leisure pairs, the specific network retained 34 positive and one negative edge among 162 estimable pairs (density = 0.216; Figure 2). The negative edge was the socializing groups–senior center edge already present among the unchanged nodes; no leisure–leisure edge was estimated. Public cultural facilities again had the largest positive-strength point estimate (1.343), followed descriptively by education/learning (1.219), clubs (1.194), socializing groups (1.052), and senior welfare centers (1.018).

Playing sports was the most connected leisure subactivity (strength = 0.758), with retained positive links to clubs (φ = 0.218), education/learning (φ = 0.147), private cultural facilities (φ = 0.137), public cultural facilities (φ = 0.130), and socializing groups (φ = 0.125). Arts participation was linked to education/learning (φ = 0.147), and social/other leisure was linked to religious activity (φ = 0.152) and socializing groups (φ = 0.107). Rest activities, despite a prevalence of 62.5%, had no retained estimable edge. Respondents named a weighted mean of 1.413 leisure categories overall (1.492 at ages 65–74 and 1.304 at ≥75; 1.401 urban and 1.449 rural; 1.438 men and 1.394 women); among leisure participants, the corresponding overall mean was 1.739.

**Figure 3:**
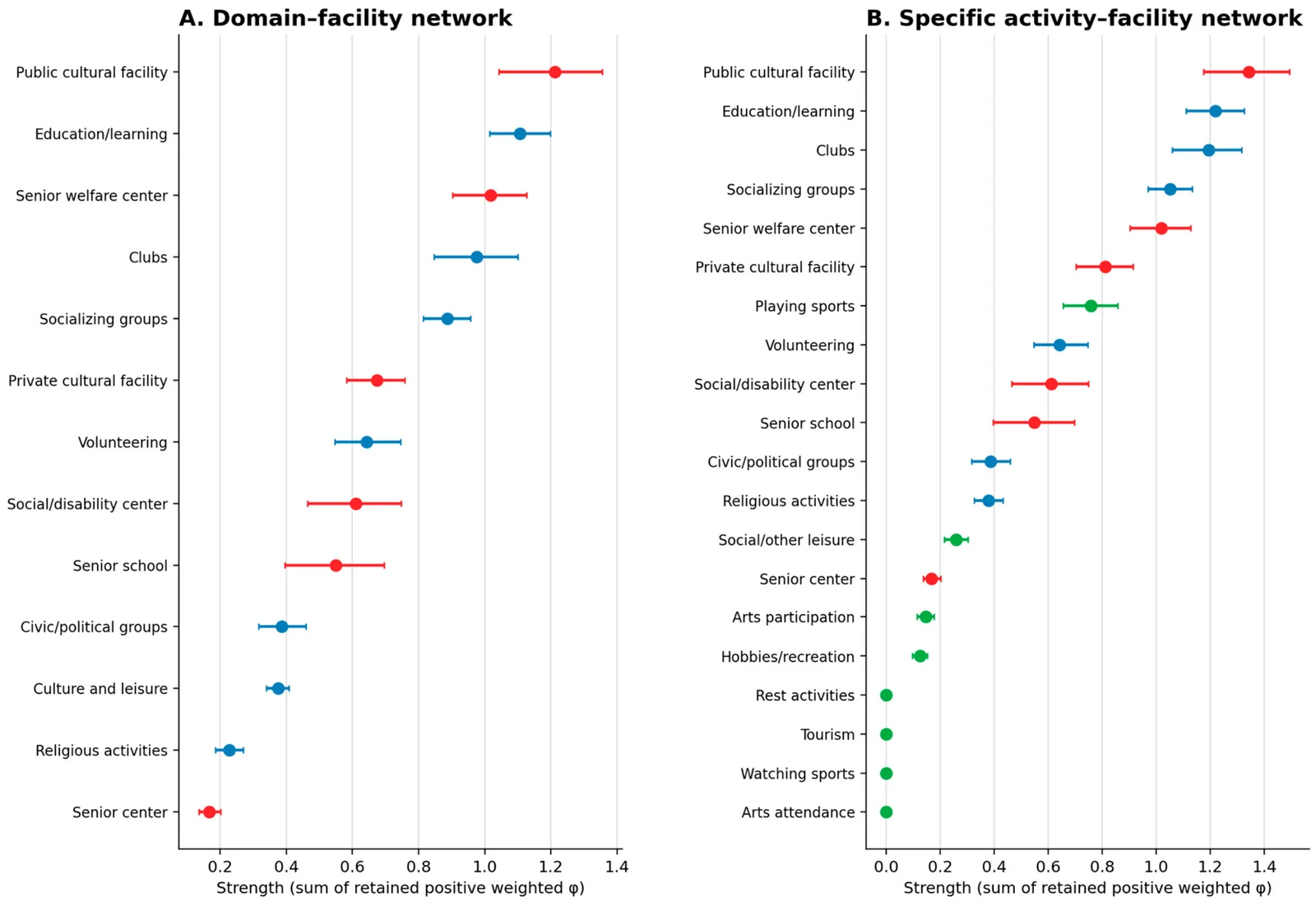
Node strength with stratified cluster-bootstrap 95% intervals. Note. Blue, green, and red points and intervals represent activity domains, leisure subactivities, and facility-use nodes, respectively. Intervals were calculated over the fixed primary network topology. Nodes with no retained positive edge have a strength of zero. Pairwise centrality differences were evaluated separately using bootstrap difference tests (Supplementary Table S10).

Node strengths and bootstrap intervals are shown in Figure 3. Positive-strength rankings were stable: median bootstrap rank correlations were 0.989 in the domain network and 0.995 in the specific network; the respective 2.5th percentiles were 0.951 and 0.986. The primary versus inclusive-threshold rank correlations were 0.973 and 0.972. In the specific network, public cultural facility strength did not differ from education/learning (difference 0.124, 95% CI −0.053 to 0.293), and education/learning did not differ from clubs (difference 0.024, 95% CI −0.106 to 0.171).

### 3.6. Subgroup Network Comparisons

**Figure 4:**
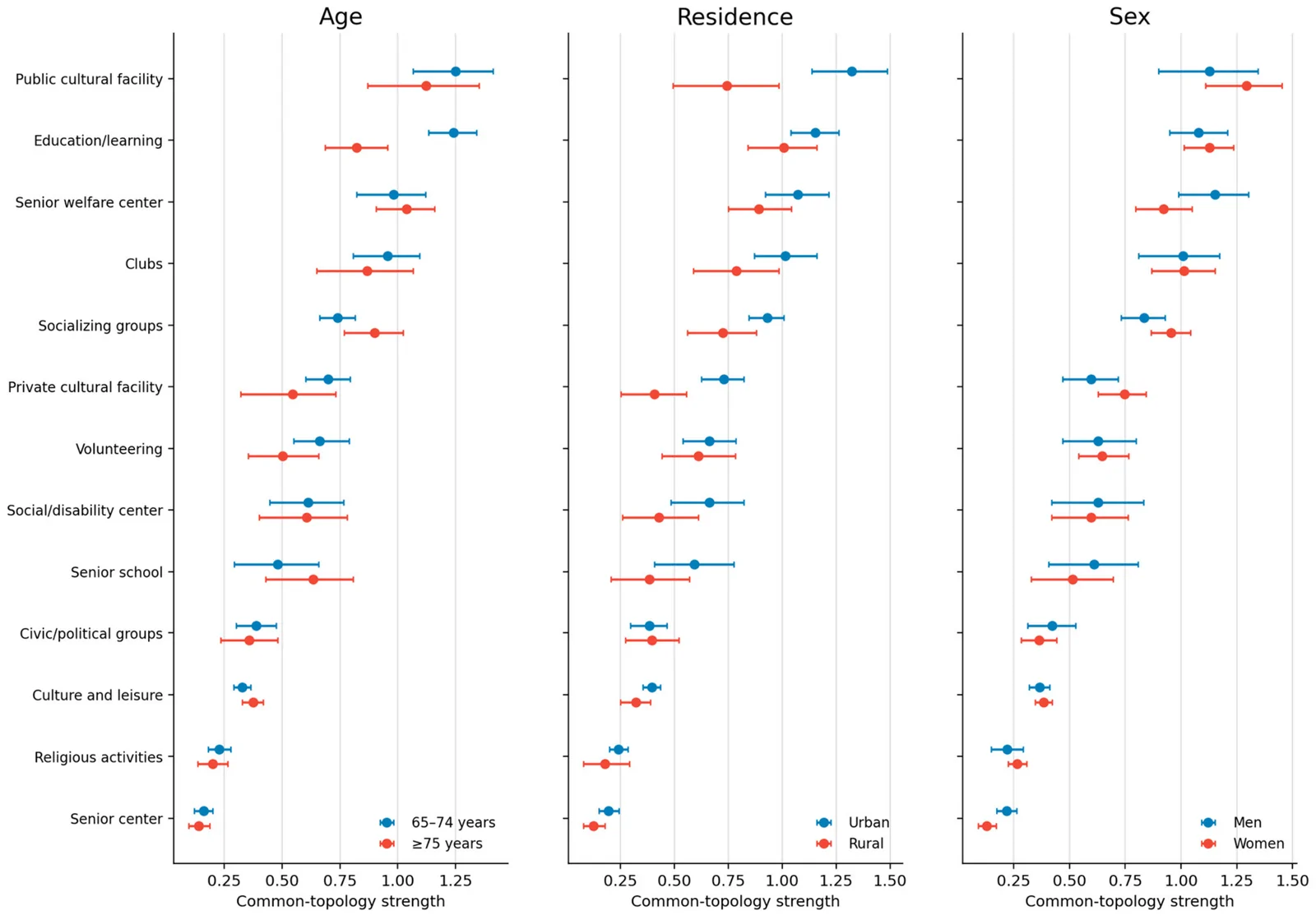
Subgroup variation in domain–facility strength. Note. Points show subgroup strength calculated on the positive topology retained in the pooled domain network; horizontal bars are stratified cluster-bootstrap 95% intervals. The common topology permits comparison of edge magnitude without changing the retained edge set between groups.

The younger-old group included 5731 participants and the older-old group 4347; 7061 lived in urban and 3017 in rural areas; 3872 were men and 6206 women. Strength-rank correlations on the common positive topology ranged from 0.901 to 0.983. Subgroup strengths are shown in Figure 4. Under the corrected differential-edge rule, the domain analyses identified 5 age, 3 residence, and 12 sex differences; the specific analyses identified 5, 6, and 10, respectively (Table 5). These counts include within-domain and facility–facility edges and should not be interpreted as equivalent numbers of activity–facility pathways.

Four age-related domain differences connected activities with facilities: education/learning was more strongly associated with public and private cultural facilities at ages 65–74, whereas socializing groups were more strongly associated with senior welfare centers and senior schools at ages ≥75 (Table 6). The alternative log-odds-ratio contrast supported the socializing–senior welfare difference (q = 0.001) but not the other three after FDR control, indicating measure sensitivity.

Urban residents had stronger domain φ links from clubs and political/social organizations to public cultural facilities. The domain public–private cultural facility edge was also stronger in urban areas, but it is a facility–facility association. None of the two activity–facility domain contrasts were significant in the log-odds-ratio sensitivity analysis after FDR control.

Among the four differential activity–facility domain edges by sex, women had stronger volunteering–public cultural facility and socializing–public/private cultural facility associations, whereas the socializing–senior center association was less negative among men. Only the socializing–senior center difference was confirmed by the log-odds-ratio contrast after FDR control. Several additional sex differences were activity–activity or facility–facility associations.

### 3.7. Sensitivity Analyses

Original and φ-limit-normalized strength rankings correlated at ρ = 0.852 in the domain network and 0.968 in the specific network. On the retained positive primary topology, the normalized domain top five were socializing groups (3.366), education/learning (2.468), clubs (2.168), public cultural facilities (2.122), and volunteering (1.991). The normalized specific-network top five were socializing groups (3.858), clubs (2.399), public cultural facilities (2.256), education/learning (2.087), and volunteering (1.991) (Supplementary Tables S4 and S7). Thus, the leading node changed in both networks: public cultural facilities moved from first to fourth in the domain network and from first to third in the specific network. The higher specific-network correlation indicates closer overall rank agreement, not preservation of the highest-ranked node. Covariate-adjusted partial correlations retained 18 of 28 primary domain edges and 22 of 35 primary specific edges with the same sign. Among the connections discussed below, education/learning–senior welfare center (adjusted r = 0.316, 95% CI 0.281–0.357) and playing sports–clubs (r = 0.187, 95% CI 0.147–0.223) were retained. Socializing groups–senior center changed from a negative primary φ to a positive adjusted r = 0.064 (95% CI 0.034–0.092), and volunteering–public cultural facility had an adjusted r = 0.092 (95% CI 0.057–0.130); neither met the |r| ≥ 0.10 retention criterion, although both had q < 0.001 (Supplementary Table S8, rows identified by these node pairs). In the targeted overlap analysis, only education/learning–senior welfare center was a retained edge among the two prespecified pairs; removing it produced rank correlations of 0.956 and 0.991 with the primary domain and specific rankings. Under the broader overlap definition, rank correlations fell to 0.797 and 0.890, and education/learning no longer ranked among the leading nodes. Thus, conclusions about the broader core were more stable than claims about education/learning specifically. The alternative subgroup log-odds-ratio contrasts supported fewer differential activity–facility edges than φ, as described above. Full edge-level results are reported in Supplementary Tables S7–S11.

## 4. Discussion

### 4.1. Principal Findings

This national analysis distinguished population reach from standardized co-participation without claiming that φ completely removes prevalence effects. Raw co-occurrence was algebraically dominated by prevalence. The φ networks highlighted public cultural facilities, education/learning, clubs, socializing groups, and senior welfare centers, and most primary edges persisted after demographic and health adjustment. However, φ-limit normalization changed the rank of public cultural facilities from first to fourth in the domain network and third in the specific network, whereas the prominence of education/learning was sensitive to removal of definitionally overlapping links; marginal constraints and item definitions therefore affected different aspects of the ordering.

The strongest cross-domain relationship connected education/learning with senior welfare center use, and education had the highest activity-side bridge strength in the primary topology. The questionnaire makes a venue interpretation plausible because senior welfare centers are explicitly listed as learning locations. This link therefore represents a reproducible co-reporting pattern but may partly double-code the same episode; it should not be interpreted as an independent behavioral pathway.

Public cultural facilities had the largest overall strength point estimate despite being used by only 7.0% of the weighted population, although several leading nodes were not statistically distinguishable. This combination of modest reach and high relational embeddedness is policy-relevant. It indicates that users of public cultural facilities disproportionately combine these settings with multiple activities and other facilities. However, the analysis cannot establish that the facility caused broader participation or that a reported activity occurred at that specific facility. The appropriate interpretation is that public cultural facility use marks a densely connected participation repertoire. In the covariate-adjusted partial-association sensitivity analysis, 18 of 28 primary domain edges and 22 of 35 primary specific edges were retained with the same sign. Facility–facility and activity–facility links were therefore interpreted separately, and the findings were not described as an Ising or conditional graphical model.

Senior centers showed the opposite pattern: high population reach but low positive φ-based strength. Their 26.5% use rate makes them important service settings, yet they had few retained positive associations at the selected threshold. The negative φ between senior center use and socializing groups does not indicate social isolation among senior-center users; 11.7% reported both. It indicates that joint occurrence was lower than expected from the two marginals. This negative association was not robust to covariate adjustment: the adjusted partial correlation was positive (r = 0.064) and below the retention threshold (Section 3.7; Supplementary Table S8). Service reach and standardized network embeddedness should therefore be reported separately.

### 4.2. Activity Repertoires and Leisure Subactivities

The specific network identified playing sports as the most connected leisure category. Because only two main leisure activities could be reported, leisure–leisure edges were not analyzed; the remaining links connect leisure categories to other activity domains or facilities. The playing sports–clubs association is also definitionally close and was included in the expanded semantic-overlap sensitivity analysis.

Rest activities were named by 62.5% of respondents but had no retained association among their 12 estimable pairs; all seven rest–other-leisure pairs were excluded. Across these 12 pairs, the maximum attainable positive φ ranged from 0.090 to 0.843, and the magnitude of the attainable negative bound ranged from 0.150 to 0.908. One pair could not attain +0.10, whereas all could attain −0.10. Using the bound corresponding to each observed association’s sign, as in Supplementary Table S2, the attainable |φ| limits ranged from 0.148 to 0.908, so all 12 pairs could reach |φ| = 0.10 in their observed direction. Nevertheless, the largest observed |φ| was only 0.065. Thus, the absence of retained rest-activity links was not explained by an unattainable threshold in the observed direction; it describes weak associations with the measured non-leisure nodes, not the importance of rest or its relations with other leisure categories. For the aggregate culture/leisure node (81.3% prevalence), the corresponding 12-pair ranges were 0.056–0.522 for the positive bound and 0.241–0.817 for the magnitude of the negative bound. Five pairs could not attain +0.10, although all could attain −0.10. The observed-sign limits ranged from 0.056 to 0.801, with five below 0.10. Culture/leisure connectivity is therefore constrained by marginal prevalences and should not be interpreted as intrinsically weak embeddedness (Supplementary Tables S2 and S4).

Volunteering also warrants attention. It was reported by only 2.5% of the weighted population but had a strength of 0.642 in both networks and a positive primary association with public cultural facility use. That link did not meet the covariate-adjusted retention threshold (r = 0.092; Section 3.7), so it should not be presented as robust to adjustment. Volunteering is frequently linked to health and mortality outcomes in longitudinal research and should not be omitted from multidomain definitions of later-life social participation [11,12].

### 4.3. Variation by Age, Residence, and Sex

High subgroup strength-rank correlations indicate a shared broad ordering, but differential-edge findings were more measure-sensitive. φ and log odds ratios answer different questions: φ is a correlation-scale association constrained by the marginals, whereas the odds ratio is invariant to the marginal distributions. Only a subset of activity–facility φ differences was supported by the log-odds-ratio sensitivity analysis.

Age-related φ differences suggested stronger learning–cultural-facility coupling at ages 65–74 and stronger socializing–older-adult-facility coupling at ages ≥75. Because only the socializing–senior welfare contrast was confirmed by the odds-ratio analysis, the other age patterns should be treated as scale-dependent exploratory findings.

Urban networks showed stronger club/civic links to public cultural facilities on the φ scale, while public–private facility coupling also differed by residence. These contrasts were not confirmed by the odds-ratio analysis after FDR control, so they may partly reflect subgroup marginals rather than different conditional pathways.

Sex contrasts included activity–activity, activity–facility, and facility–facility edges. The socializing–senior center contrast was robust to the odds-ratio analysis; most other φ differences were not. The binary sex variable and unmeasured compositional differences limit interpretation.

### 4.4. Implications for Geriatric Practice and Community Policy

Social participation has been associated with disability, frailty, cognitive function, dementia, and well-being outcomes, although the present analysis did not test health outcomes [11,12,13,14,27].

The structural results suggest three practical distinctions. First, high-reach settings such as senior centers may require program diversification to create stronger links with learning, volunteering, or cultural participation. For example, senior centers could offer introductory learning and arts activities or volunteering sessions in partnership with senior welfare centers and public cultural facilities; shared schedules and referrals could then connect interested participants with community classes, clubs, or volunteer groups. These are program options for future evaluation, not effects established by these cross-sectional data. Second, highly embedded but lower-reach settings such as public cultural facilities may be candidate partners for linkage-based programs, although roughly half of this node’s network strength reflects co-use of other facilities rather than activity–facility links, and the cross-sectional design cannot show that facility use extends participation. Third, education/learning appears to be a potentially scalable connector across welfare and cultural settings. Interventions could therefore be designed as linked participation pathways rather than isolated programs—for example, a welfare-center learning course that transitions into a club, volunteer role, or community cultural program.

These are system-level hypotheses rather than causal recommendations. A node with high strength is not automatically the best intervention target, and centrality does not show what would happen if a program were expanded. Prospective evaluations should test whether deliberate linkage between activities and facilities increases sustained participation and whether effects differ by age, sex, health, mobility, rurality, or socioeconomic status [28,29].

### 4.5. Strengths and Limitations

Strengths of this study include a large national sample, explicit survey weighting, design-aware cluster bootstrap, separation of aggregate and component leisure nodes, exclusion of structurally coupled leisure–leisure pairs, attainable-φ and normalized-φ analyses, covariate-adjusted partial correlations, alternative odds-ratio contrasts, and centrality difference tests. Exact item wording and complete reproducible code are supplied.

Several limitations should be considered. First, cross-sectional associations cannot establish temporal order or causality. Second, these are within-person co-participation networks, not interpersonal social networks or conditional graphical models. Third, self-reported facility use does not identify where each activity occurred. Fourth, some items can double-code the same episode; the expanded overlap analysis materially reduced the centrality of education/learning. Fifth, φ mitigates but does not eliminate marginal-prevalence constraints, and the |φ| threshold is not equally attainable for all pairs. Sixth, the two-response cap required exclusion of all leisure–leisure pairs. Seventh, subgroup differences were exploratory and several φ contrasts were not supported by marginal-invariant log-odds-ratio comparisons. Eighth, the public file lacked the original stratum identifier; 30 observed province × urban/rural strata and 977 survey areas approximated the sampling design. Finally, no health outcome was modeled, so practical implications remain hypotheses for longitudinal or program-evaluation research. Because the sample comprised community-dwelling older adults, the findings may not generalize to older adults living in nursing homes or other institutional settings, where participation opportunities and facility access may differ.

## 5. Conclusions

In a nationally representative sample of older Koreans, public cultural facilities and several organized activities occupied prominent positions in prevalence-aware co-participation networks. Positive-strength ranks showed high agreement across bootstrap samples on the fixed primary topology. Agreement with φ-limit-normalized ranks was higher in the specific network (ρ = 0.968) than in the domain network (ρ = 0.852), but socializing groups replaced public cultural facilities as the leading node in both; public cultural facilities moved to third and fourth, respectively. Item overlap and alternative association measures also changed some specific conclusions. The results should guide cautious hypothesis generation about activity–facility ecosystems, not causal or prevalence-free claims.

## Figures and Tables

**Table 1:** Participant characteristics.

Characteristic	Category	*n*	Weighted %
Sex			
	Men	3872	43.9
	Women	6206	56.1
Age group, years			
	65–69	3249	34.5
	70–74	2482	23.6
	75–79	1950	17.3
	80–84	1544	14.0
	85–89	668	8.2
	≥90	185	2.5
Residence			
	Urban (dong)	7061	73.9
	Rural (eup/myeon)	3017	26.1
Marital status			
	With spouse	5951	60.0
	Without spouse	4127	40.0
Educational attainment			
	No formal education	1488	12.3
	Elementary school	2961	28.2
	Middle school	2127	21.2
	High school	2876	31.2
	College or higher	626	7.0
Employment history			
	Currently employed	3948	39.0
	Previously employed	4773	47.1
	Never employed	1357	13.9
Household income quintile			
	Q1	2132	20.0
	Q2	2205	20.0
	Q3	2057	20.0
	Q4	1925	20.0
	Q5	1759	20.0
Self-rated health			
	Very good	240	2.7
	Good	3873	40.0
	Fair	3445	34.2
	Poor	2107	19.2
	Very poor	286	2.6
	Missing/not applicable	127	1.2

Note. *N* = 10,078. Counts are unweighted; percentages use the standardized survey weights. The not-applicable code for self-rated health is shown as missing/not applicable.

**Table 2:** Weighted prevalence of activity and facility nodes.

Node Group	Node	*n*	Weighted %
Activity domain			
	Culture and leisure	8295	81.3
	Education/learning	1397	13.3
	Clubs	634	6.6
	Socializing groups	5301	54.2
	Civic/political groups	144	1.3
	Volunteering	269	2.5
	Religious activities	3416	33.1
Leisure subactivity			
	Arts attendance	150	1.5
	Arts participation	247	2.2
	Watching sports	181	2.2
	Playing sports	696	7.4
	Tourism	344	3.5
	Hobbies/recreation	2847	29.1
	Rest activities	6410	62.5
	Social/other leisure	3590	33.0
Facility			
	Senior center	2848	26.5
	Senior welfare center	957	9.6
	Social/disability center	307	3.5
	Senior school	155	1.7
	Public cultural facility	629	7.0
	Private cultural facility	333	3.5

Note. Counts are unweighted; percentages are survey-weighted. Leisure subactivity percentages refer to categories named among respondents’ two main leisure activities.

**Table 3:** Network structure, prevalence dependence, and stability.

Network Characteristic	Domain–Facility	Specific Activity–Facility
Nodes	13	20
Positive/negative edges	27/1	34/1
Density	0.359	0.216
Prevalence vs. φ-based strength, ρ	−0.077	0.158
Median bootstrap strength-rank correlation, ρ	0.989	0.995
Primary vs. inclusive-threshold strength-rank correlation, ρ	0.973	0.972

Note. Edges +/− satisfy BH-FDR q < 0.05, a percentile 95% bootstrap interval excluding zero, and |φ| ≥ 0.10. The specific network excludes all 28 leisure–leisure pairs; its density denominator is therefore 162. Bootstrap rank ρ is the median correlation between original and bootstrap positive-strength ranks. Threshold rank ρ compares the primary and inclusive |φ| ≥ 0.05 networks.

**Table 4:** Highest positive-strength nodes in each network.

Node	Degree	Strength (95% CI)	Bridge Strength (95% CI)
Domain–facility network			
Public cultural facility	8	1.213 (1.043–1.355)	0.582 (0.492–0.666)
Education/learning	6	1.107 (1.015–1.198)	0.712 (0.645–0.779)
Senior welfare center	5	1.018 (0.903–1.126)	0.309 (0.271–0.350)
Clubs	6	0.976 (0.847–1.100)	0.271 (0.191–0.345)
Socializing groups	6	0.888 (0.814–0.957)	0.251 (0.209–0.286)
Specific activity–facility network			
Public cultural facility	9	1.343 (1.174–1.492)	0.712 (0.604–0.814)
Education/learning	7	1.219 (1.109–1.324)	0.712 (0.645–0.779)
Clubs	7	1.194 (1.059–1.316)	0.271 (0.191–0.345)
Socializing groups	8	1.052 (0.969–1.134)	0.251 (0.209–0.286)
Senior welfare center	5	1.018 (0.903–1.126)	0.309 (0.271–0.350)

Note. Strength is the sum of retained positive weighted φ coefficients. Bridge strength is the sum of retained positive activity–facility coefficients. Complete results are provided in the Supplementary Material.

**Table 5:** Similarity and differential-edge counts in subgroup comparisons.

Network	Groups, *n* (A/B)	Retained Edges (A/B)	Edge-Weight ρ	Strength-Rank ρ	Differential Edges (all/A–F)
Age (65–74 vs. ≥75 years)					
Domain–facility	5731/4347	25/30	0.683	0.907	5/4
Specific	5731/4347	32/35	0.753	0.958	5/5
Residence (urban vs. rural)					
Domain–facility	7061/3017	31/19	0.764	0.934	3/2
Specific	7061/3017	40/22	0.670	0.967	6/4
Sex (men vs. women)					
Domain–facility	3872/6206	31/29	0.736	0.901	12/4
Specific	3872/6206	36/35	0.756	0.983	10/3

Note. Groups are ordered A/B as named. Retained counts use the full primary rule within each subgroup. Differential edges meet the primary rule in at least one subgroup, |Δφ| ≥ 0.05, BH-FDR q < 0.05, and a percentile interval excluding zero. The specific network excludes leisure–leisure pairs from estimation and FDR correction. A–F = activity–facility.

**Table 6:** Differential activity–facility edges in the domain network.

Edge	Group A φ	Group B φ	Δφ (95% CI)	FDR q
Age (A: 65–74; B: ≥75 years)				
Education/learning–Public cultural facility	0.217	0.118	0.100 (0.039 to 0.156)	0.023
Education/learning–Private cultural facility	0.273	0.090	0.183 (0.108 to 0.256)	<0.001
Socializing groups–Senior welfare center	−0.006	0.116	−0.122 (−0.172 to −0.074)	<0.001
Socializing groups–Senior school	0.021	0.110	−0.089 (−0.136 to −0.033)	0.014
Residence (A: urban; B: rural)				
Clubs–Public cultural facility	0.164	0.047	0.117 (0.045 to 0.185)	0.027
Civic/political groups–Public cultural facility	0.102	−0.006	0.109 (0.055 to 0.165)	0.008
Sex (A: men; B: women)				
Socializing groups–Senior center	−0.039	−0.146	0.106 (0.060 to 0.148)	<0.001
Socializing groups–Public cultural facility	0.115	0.167	−0.052 (−0.091 to −0.014)	0.043
Socializing groups–Private cultural facility	0.075	0.138	−0.063 (−0.107 to −0.021)	0.029
Volunteering–Public cultural facility	0.049	0.146	−0.097 (−0.165 to −0.024)	0.033

Note. Δφ = group A minus group B. Positive values indicate a stronger association in group A. Only activity–facility edges meeting all differential-edge criteria are shown. FDR q values were adjusted within each contrast and network.

## Data Availability

The deidentified public-use dataset, questionnaire, codebook, and report for the 2023 National Survey of Older Koreans are available through the Ministry of Health and Welfare: https://www.mohw.go.kr/board.es?act=view&bid=0019&list_no=1483359&mid=a10411010100 (accessed on 24 September 2026). The recoded binary analysis data can be regenerated from the public SAV file using Supplementary Code S1.

## Supplementary Materials

The following supporting information can be downloaded at https://www.mdpi.com/article/10.3390/geriatrics11050150/s1, The supplementary tables are provided as worksheets in the accompanying Excel workbook. Table S1: Node Codebook; Table S2: Pooled Edges; Table S3A: Domain Matrix; Table S3B: Specific Matrix; Table S4: Centrality; Table S5: Subgroup Edges; Table S6: Subgroup Centrality; Table S7: Phi-limit Sensitivity; Table S8: Adjusted Partial; Table S9: Semantic Overlap; Table S10: Centrality Differences; Table S11: Leisure Count; Supplementary Code S1: Reproducible scripts and README.
